# Antioxidant Activity of Caffeic Acid against Iron-Induced Free Radical Generation—A Chemical Approach

**DOI:** 10.1371/journal.pone.0129963

**Published:** 2015-06-22

**Authors:** Thiago C. Genaro-Mattos, Ângelo Q. Maurício, Daniel Rettori, Antonio Alonso, Marcelo Hermes-Lima

**Affiliations:** 1 Laboratório de Radicais Livres, Departamento de Biologia Celular, Universidade de Brasília, Brasília, DF, Brazil; 2 Laboratório de Espectrometria de Massa, Embrapa Recursos Genéticos e Biotecnologia, Brasília, DF, Brazil; 3 Instituto de Química, Universidade de Brasília, Brasília, DF, Brazil; 4 Laboratório de Química e Bioquímica de Espécies Altamente Reativas, Departamento de Ciências Exatas e da Terra, Universidade Federal de São Paulo–UNIFESP, São Paulo, SP, Brazil; 5 Instituto de Física, Universidade Federal de Goiás, Goiânia, GO, Brazil; Case Western Reserve University, UNITED STATES

## Abstract

Caffeic acid (CA) is a phenolic compound widely found in coffee beans with known beneficial effects *in vivo*. Many studies showed that CA has anti-inflammatory, anti-mutagenic, antibacterial and anti-carcinogenic properties, which could be linked to its antioxidant activity. Taking in consideration the reported *in vitro* antioxidant mechanism of other polyphenols, our working hypothesis was that the CA antioxidant activity could be related to its metal-chelating property. With that in mind, we sought to investigate the chemical antioxidant mechanism of CA against *in vitro* iron-induced oxidative damage under different assay conditions. CA was able to prevent hydroxyl radical formation promoted by the classical Fenton reaction, as determined by 2-deoxyribose (2-DR) oxidative degradation and DMPO hydroxylation. In addition to its ability to prevent hydroxyl radical formation, CA had a great inhibition of membrane lipid peroxidation. In the lipid peroxidation assays CA acted as both metal-chelator and as hydrogen donor, preventing the deleterious action promoted by lipid-derived peroxyl and alkoxyl radicals. Our results indicate that the observed antioxidant effects were mostly due to the formation of iron-CA complexes, which are able to prevent 2-DR oxidation and DMPO hydroxylation. Noteworthy, the formation of iron-CA complexes and prevention of oxidative damage was directly related to the pH of the medium, showing better antioxidant activity at higher pH values. Moreover, in the presence of lipid membranes the antioxidant potency of CA was much higher, indicating its enhanced effectiveness in a hydrophobic environment. Overall, our results show that CA acts as an antioxidant through an iron chelating mechanism, preventing the formation of free hydroxyl radicals and, therefore, inhibiting Fenton-induced oxidative damage. The chemical properties of CA described here—in association with its reported signaling effects—could be an explanation to its beneficial effects observed *in vivo*.

## Introduction

Caffeic acid (CA) is a phenolic compound produced by the secondary metabolism of plants and is the major hydroxycinnamic acid present in the human diet [1]. It is commonly found in several fruits and coffee beans, one of the major commodities consumed in the western diet [1]. Clifford (2000) reported that the absorption of this compound is directly associated with the amount of coffee consumed, being able to achieve 500 to 800 mg/day in individuals with high coffee intake [2]. In addition, several studies demonstrated that CA is absorbed in rat [3] and human intestines [4,5], where 95% of total CA ingested was absorbed. Several other studies showed that plasmatic concentrations of polyphenols–including CA–are between 0.3 and 1.5 μM [6–9].

The antioxidant activity of CA was previously studied by different research groups [9,10,11,12]. For instance, Nardini et al. reported that CA inhibited, in a dose-dependent manner, human LDL lipoperoxidation induced by cupric ions [10]. The authors show that CA is able to reduce lipoperoxyl radicals (ROO^•^)–by donating a hydrogen atom–to its corresponding hydroperoxide, which inhibits the lipid peroxidation chain reaction. In addition to its antioxidant capacity, studies have shown that CA has anti-inflammatory [11], anti-mutagenic [12], antibacterial [13] and anti-carcinogenic properties [14].

Several detailed studies were conducted to determine the ability of CA, and its metabolites, to bind metal ions and to study their influence on redox reactions mediated by these metals [15–17]. Moon and Terao (1998) reported the antioxidant activity of CA and dihydrocaffeic acid (H_2_CA) on human LDL oxidation induced by copper ions, where both antioxidants extended the lipoperoxidation initiation phase [18]. Moreover, the authors saw that CA and H_2_CA inhibited the formation of methyl linoleate hydroperoxides induced by the azo-iniatiator AMVN (2.2’-Azobis-2.4-dimethylvaleronitrile) [18]. Noteworthy, H_2_CA was detected in human plasma as a cathecol metabolite of CA [19].

Despite the numerous studies reporting the antioxidant properties of CA, the exact antioxidant mechanism in systems containing iron ions remains unclear. Thus, this study was conducted to characterize the ability of CA to complex iron ions as well as the antioxidant activity of this complex in several oxidant systems. Briefly, we observed that CA is able to complex Fe(II) ions and to inhibit *in vitro* DMPO hydroxylation (an EPR assay), 2-deoxyribose (2-DR) oxidation (both are assays for hydroxyl radical formation) and lipid peroxidation of rat liver membranes. Our results also show that the antioxidant activity is dependent on the medium pH and on the concentrations of CA and iron. Our results, overall, suggest that CA has a chelating-antioxidant mechanism.

## Materials and Methods

### Chemicals

Caffeic acid, 2-deoxyribose (2-DR), potassium phosphate, 5,5-Dimethyl-1-Pyrroline-N-Oxide (DMPO) and thiobarbituric acid (TBA) were purchased from Sigma. Ferric chloride, H_2_O_2_ and ferrous ammonium sulfate were from Merck. Other reagents were of analytical grade. Phosphate buffer (pH 7.2) and solutions of 2-DR were prepared weekly, while solutions of metal chelators (0.5 mM), Fe(II) (0.5 mM) and H_2_O_2_ (1 mM) were prepared prior to use. Deionized water (Millipore; ρ ≥ 18 MΩ cm) was used for all solutions. DMPO was purified prior to the experiments. TBA solutions (1% w/v) were prepared daily in 50 mM NaOH.

### Animal care and ethics statement

Experiments were performed at University of Brasilia. Two adult male Wistar rats were provided by Dr. Egle de Almeida’s Laboratory located at the University of Brasilia, Brazil. They were housed individually in stainless steel cages for two days under controlled conditions (21–23°C, 12 hour light/dark cycles) and were allowed water and food *ad libitum*. Rats were then sacrificed by cervical dislocation and livers were excised, washed in a cold 0.9% NaCl solution, rapidly frozen in liquid nitrogen and stored at −80°C for further analyses. This protocol was approved by the Ethics Committee for the Use of Animals of the Institute of Biological Sciences, University of Brasilia, Brazil as UnB-DOC 120380/2009.

### Spectrophotometric determinations of Fe(II)-CA complexes

Analyses were carried out in a Hitachi U-2001 spectrophotometer using a 10 mm path-length cuvette. Reactions were carried out in the presence of 10 mM phosphate buffer (pH 7.2), CA and Fe(II). Spectra were obtained immediately after the addition of iron. Absorbances were subtracted from respective blanks.

### 2-Deoxyribose oxidation

The method is based on the determination of malondialdehyde (MDA) [20,21], a degradation product of 2-DR oxidation. Typical reactions were started by the addition of Fe(II) to solutions (0.5 mL final volume) containing 5 mM 2-DR, 10 mM phosphate (pH 7.2), CA and H_2_O_2_. Reactions were carried out for 10 min at 25 ± 1°C and stopped by the addition of 0.5 mL 4% phosphoric acid plus 0.5 mL TBA solution. After boiling (15 min), absorbances were measured at 532 nm [21].

### Electron Paramagnetic Resonance

EPR spectra were obtained with a Bruker-EMX spectrometer equipped with the standard (ER4102) rectangular resonator operating in the TE_102_ mode. Samples of the mixtures were transferred to capillary tubes (volume, 50 μL) and measured at room temperature. Instrument settings: microwave frequency, 9.42 GHz; microwave power, 10 mW; field-modulation frequency, 100 kHz; field-modulation amplitude, 1.0 G; receiver gain, 1.25 x 10^5^; time constant, 41 ms; scan rate, 2.5 G s^-1^; number of scans: 3. Spectra were registered 3 min after Fe(II) addition to solutions containing 10 mM phosphate (pH 7.2), 50 mM DMPO in the presence or absence of CA.

### Lipid Peroxidation

Analyses were done using total rat liver homogenate and quantifying TBARS. Livers from two Wistar rats were homogenized with cold phosphate buffer (50 mM plus KCl 125 mM) in a 1:4 w/v ratio. This mixture was centrifuged at 1000 rpm for 15 min at 4°C, then the supernatant was used for assays. Typical reactions were started by addition of Fe(II) to solutions (0.5 mL final volume) containing 10 mM phosphate (pH 7.2), 5% v/v rat liver homogenate, KCl 125 mM, CA and H_2_O_2._ Reactions were performed at 24–25°C and stopped by 0.2 mL 7% phosphoric acid plus 0.2 mL TBA solution. After boiling for 15 min, solutions were cooled and absorbance values were measured at 532 and 600 nm. The final “readings” were obtained by subtracting A_532_ from A_600_.

## Results

### Spectrophotometric analyses of Fe(II)-CA complex

**Fig 1:**
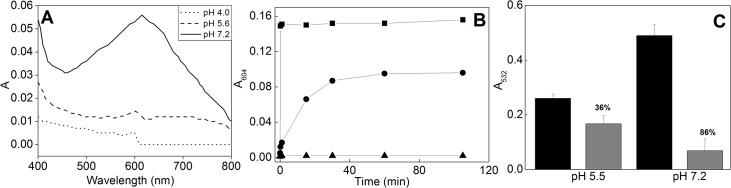
Spectroscopic analyses of CA. **(A)** Visible spectra at different pHs for mixtures of CA and Fe(II). Experimental conditions: 8 mM Hepes/MES; 8 μM Fe(II); 80 μM CA; figure shows representative spectra (n = 3). **(B)** Time course of CA and Fe(II) complexation at pHs 7.2 (■), 5.6 (●) and 4.0 (▲). Experimental conditions: 20 mM Hepes/MES; 30 μM Fe(II); 200 μM CA; figure shows representative traces (n = 3). **(C)** Effect of pH on 2-DR degradation induced by Fenton reagents in the absence (*dark bars*) or presence of CA (*light bars*). Experimental conditions: 20 mM Hepes/Mes; 10 mM 2-DR; 30 μM Fe(II); 100 μM H_2_O_2_; 200 μM CA. n = 9.

The formation of iron-CA complex can be observed in 604 nm and it is directly associated with its ability to bind iron ions through the catechol moiety [22]. CA was incubated with Fe(II) in different buffered media (at pHs 4.0, 5.5 and 7.2) to test the pH influence on the complex formation. Fig 1A shows that complexation is influenced by pH, with higher complex-formation values at pH 7.2 than at 4.0 or 5.5. The higher the absorbance values, the higher the metal complexation. In order to determine the complexation rate, a kinetic study was also performed (Fig 1B). The formation of the complex was monitored at 604 nm and the absorbance values were recorded until 105 min. There is no effect at pH 4.0, suggesting that CA does not complex Fe(II) through the cathecol moiety. At pH 5.5 the complexation is detectable within 1 min and reaches saturation after 30 min, indicating that the reaction reached the equilibrium. At pH 7.2 the complexation reaches its maximum within 10 sec, remaining unchanged through the entire incubation, indicating that the complexation through the catechol moiety happens very quickly at this pH.

### Influence of the pH on the 2-DR damage

After characterizing the complex formation we performed an experiment to test the pH influence on the antioxidant capacity of CA. Fig 1C shows that the antioxidant effect of CA against 2-DR damage–induced by Fenton reagents—is pH dependent. At pH 5.5 the antioxidant efficiency afforded by CA was only 36%, much lower than at pH 7.2 (86% protection).

### Effect of CA concentration

**Fig 2:**
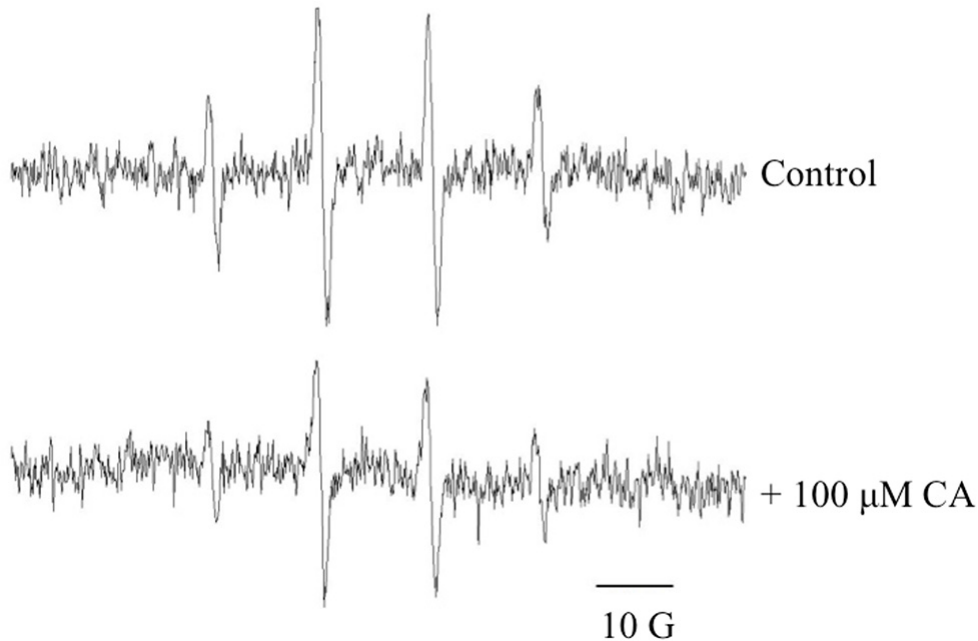
Typical EPR signal of DMPO-OH adduct induced by Fenton reagents. Signal obtained in the absence of CA (*upper signal*) and in the presence of 100 μM CA (*bottom signal*). Experimental conditions: 10 mM KPi (pH 7.2); 20 mM DMPO; 100 μM H_2_O_2_; 50 μM Fe(II). Signal is representative of three independent experiments;

**Fig 3:**
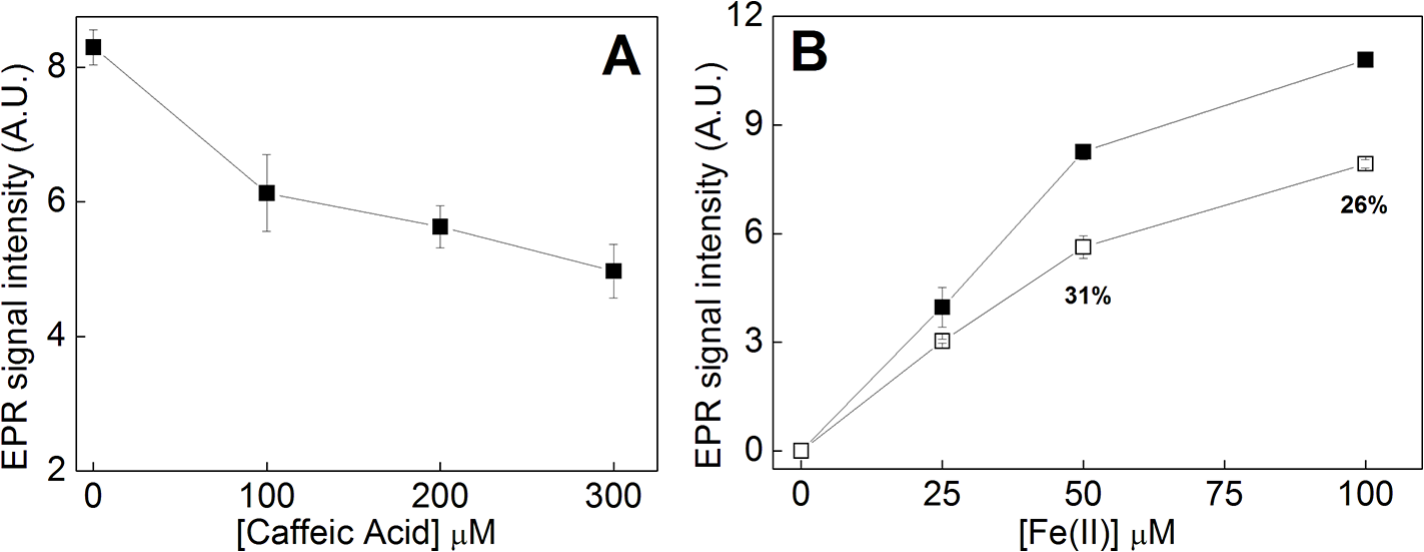
EPR determinations of DMPO hydroxylation induced by Fenton reagents. **(A)** Effect of CA on DMPO hydroxylation. Experimental conditions: 10 mM KPi (pH 7.2); 20 mM DMPO; 100 μM H_2_O_2_; 50 μM Fe(II); 0–300 μM CA. n = 3; **(B)** Effect of Fe(II) on DMPO hydroxylation in the absence (*solid squares*) or presence (*open squares*) of CA. Experimental conditions: 10 mM KPi; 20 mM DMPO; 100 μM H_2_O_2_; 200 μM CA; 0–100 μM Fe(II). n = 3.

**Fig 4:**
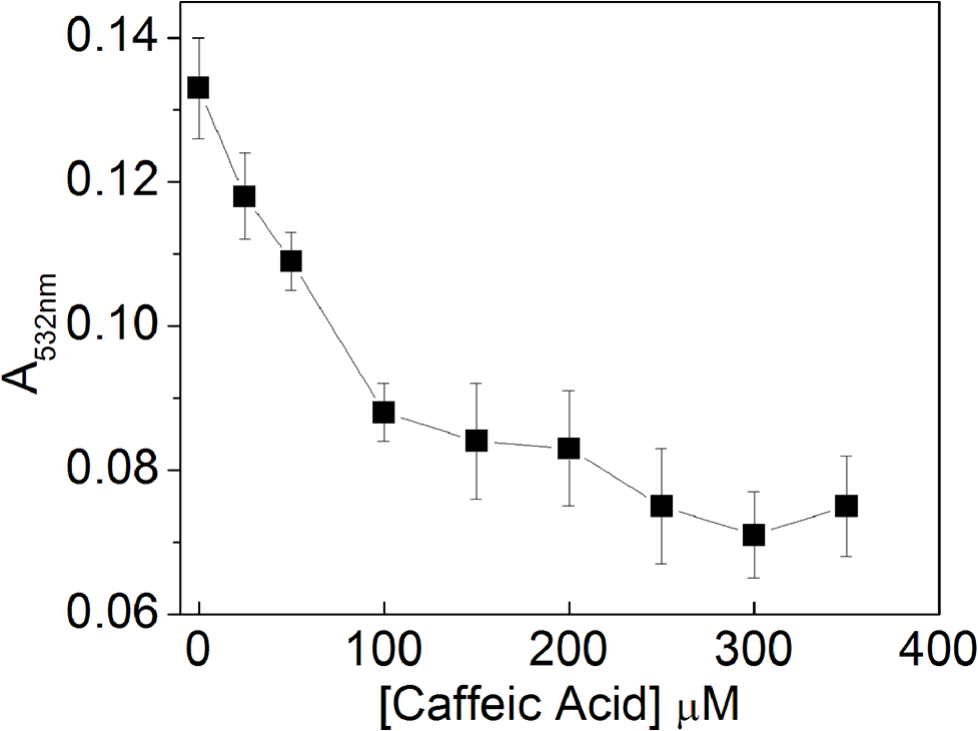
Effect of CA on 2-DR oxidative degradation induced by Fenton reagents. Experimental conditions: 10 mM KP (pH 7.2)i; 5 mM 2-DR; 100 μM H_2_O_2_; 50 μM Fe(II); 0–350 μM CA. n = 3. The angular coefficient changes at 100 μM CA, suggesting a 1:2 Fe(II):CA complexation ratio.

The effect of CA on hydroxyl radical formation from Fenton reagents (H_2_O_2_ 100 μM plus Fe(II) 50 μM) was determined employing the EPR assay of DMPO hydroxylation (Fig 2). The antioxidant action of CA was concentration dependent, with a two-phase inhibitory profile and maximum inhibitory action happed from 0 to 0.1 mM CA (Fig 3A). A similar result—employing the same concentrations of Fenton reagents—was observed when the CA titration was determined using the 2-DR assay (Fig 4). For both assays, 0.2 mM CA presented a near-maximal inhibitory effect.

### Effect of iron concentration

CA also inhibited DMPO hydroxylation, induced by Fenton reagents, with different concentrations of iron. Moreover, in the presence of 50 μM Fe(II), CA inhibited EPR signal by 31% (Fig 3B). A comparison experiment with TA (a polyphenol with 25 free hydroxyl groups, possessing a better environment for iron chelation and/or hydroxyl radical scavenging activity[23]) showed the same profile with the increase in Fe(II) concentration, except the percent of protection was increased to 53% (data not shown). In addition, the effect of iron concentration on CA antioxidant action was also examined using the 2-DR assay. It is observed that an augment in Fe(II) concentration is accompanied by a decrease in the antioxidant efficiency of CA (referred as percentage at Fig 3B).

### Effect of 2-DR concentration

In order to characterize the antioxidant mechanism of CA we performed a competition experiment in the presence of different 2-DR concentrations (Table 1). Briefly, this experiment consists in increasing the hydroxyl radical target molecule (2-DR in this case) and monitors the percent of inhibition. If CA and 2-DR shall compete for the hydroxyl radical, the inhibition performed by CA would decrease with the increase in 2-DR concentration, suggesting a scavenger antioxidant molecule. It can be seen that CA prevented 2-DR oxidation in all experimental conditions. In addition, the raise in 2-DR concentration did not influence the relative CA effect, since in all conditions the percent of inhibition remained unchanged.

**Table 1:** Effect of 2-DR concentration in the 2-DR oxidation assay.

[2-DR] mM	Experimental conditions		
	*A*_*532*_ *without CA*	*A*_*532*_ *with CA*	*% of protection*
**0**	0.002 ± 0.004	0.000 ± 0.003	—-
**5**	0.125 ± 0.028	0.100 ± 0.031	20.5%
**10**	0.158 ± 0.040	0.127 ± 0.034	19.4%
**20**	0.173 ± 0.030	0.135 ± 0.041	22.0%

Experimental conditions: 10 mM KPi (pH 7.2), 30 μM Fe(II) (or Fe(III) for the blanks), 100 μM H_2_O_2_, 200 μM CA and 0 to 20 mM 2-DR. The reaction was incubated for 10 min after addition of Fe(II). n = 6. Absorbance values were corrected for the effect of Fe(III)-induced 2-DR oxidation (see [19]).

### Effect of CA on lipid peroxidation

**Fig 5:**
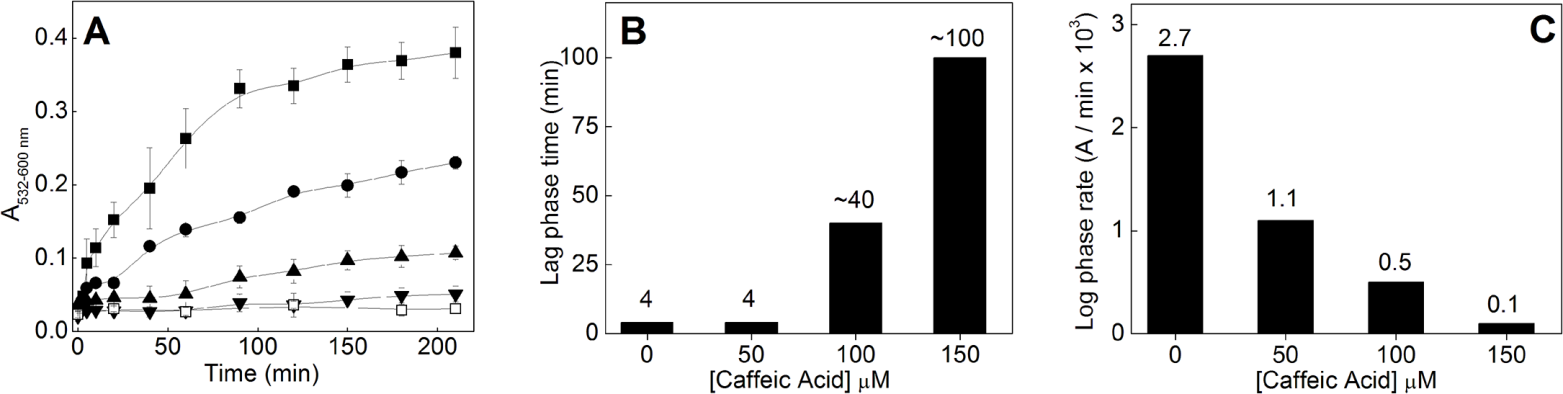
Effect of caffeic acid on lipid peroxidation. **(A)** Effect of CA on the *in vitro* rat liver peroxidation induced by Fenton reagents. Experimental conditions: 10 mM KPi (pH 7.2); 5% v/v liver homogenate; 100 μM H_2_O_2_; 50 μM Fe(II) (■); Fe(II) plus 50 μM CA (●); Fe(II) plus 100 μM CA (▲); Fe plus 150 μM CA (▼); Fe plus 100 μM BHT (□). n = 9; **(B)** Effect of CA on the lag phase of lipid peroxidation. **(C)** Effect of CA on the log phase of peroxidation—experimental conditions are the same as in panel-A.

CA antioxidant effect was also analyzed against lipid peroxidation. Hepatic membranes were incubated for 3.5 h with Fenton reagents in the absence and presence of different CA concentrations (Fig 5A). CA inhibited lipid peroxidation in a dose-dependent manner, and at 150 μM reached a similar efficiency of 100 μM BHT (a well-known chain breaking antioxidant). We also analyzed the effect of CA concentration on the peroxidation lag phase (Fig 5B). The lag phase of control reaction was 4 min, being expanded 10 and 25 times with 100 μM and 150 μM CA, respectively. The propagation phase of the peroxidation reaction (also known as the log phase) had its rate decreased from 2.7 x 10^−3^ A/min to 0.5 x 10^−3^ A/min at 100 μM, and to approximately 0.1 x 10^−3^ A/min at 150 μM (Fig 5C). In addition, CA inhibited TBARS formation after 3 h incubation by 72 and 87% when employed at 100 and 150 μM, respectively (Fig 4A). 50 μM CA had no effect on the lag-phase, but decreased the rate of the log phase by 59% and the levels of peroxidation (at 3 h incubation) by 41%.

## Discussion

**Fig 6:**
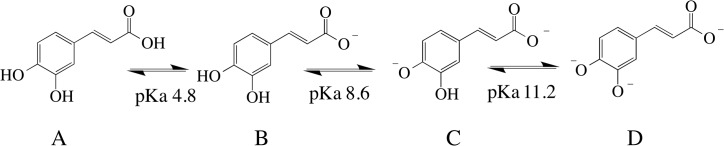
Deprotonation of caffeic acid and the respective pKa values.

The pH influence on the complex formation (Fig 1A) is related to CA’s three ionizable hydroxyl groups, with pKa values of 4.8, 8.6 and 11.2 (carboxylic group, *p*-hydroxy and *m*-hydroxy hydrogens, respectively) [24]. The increase in the medium pH (from 4.0 to 7.2) leads to an enhancement on the dissociation of these groups and, consequently, the ability of CA to chelate iron is also increased (which is also observed in the kinetic experiment–Fig 1B). The lack of complexation through the cathecol moiety at pH 4.0 is due to the fact that such pH is lower than the pKa of the carboxylic group and a negligible amount of the cathecol groups is deprotonated (see pKa values at Fig 6), making the complexation an unfavorable process.

At pH 5.5 the carboxylate ion (situation B at Fig 6) is the major species present in solution and the equilibrium also involves the deprotonation of the second hydroxyl group (*p*-hydroxy). This situation would enhance the ability of CA to bind iron ions [24]. The increase in the pH to 7.2 favors even more the ionization of the *p*-hydroxyl group, once the pH is closer to its pKa value. Moreover, the fast complexation at pH 7.2 (within 10 sec) may be due to the initial binding between Fe(II) and the *p*-hydroxyl group, enhancing the electronic density over the aromatic ring and stabilizing the complex. This could lower the pKa of the other hydroxyl group (*m*-hydroxyl) and facilitate the second bond between CA and Fe(II). Pardo-Andreu et al. (2006) made a similar proposal for mangiferin—a molecule bearing a cathecol moiety and several other hydroxyl groups—during iron complexation [25]. Furthermore, Kunsági-Mati et al. (2008) reported the formation of a thermodynamic favorable complex (ΔG^0^ = -18.6 kJ/mol) with a 1:1 Fe(II):CA ratio at pH 3.2, in which is suggested that CA binds Fe(II) through the carboxylate moiety [26]. It is worth mentioning that we did not observe the complexation between CA and Fe(II) at pH 4.0 because we were monitoring it through the absorbance of cathecol moiety. In addition to the 1:1 ratio, CA may complex iron ions with other stoichiometries, such as 1:2 and 1:3 Fe(II):CA, which is pH-dependent and is expected to have higher antioxidant effect [27]. Indeed, the antioxidant activity of CA is influenced by the medium pH, with an increase in its efficiency with the augment in the pH (see Fig 1C).

As shown in Fig 3A, the titration of CA has a two-phase antioxidant profile (the same behavior is observed employing the 2-DR assay–Fig 4). Fe(II) was present in 50 μM in both experiments and the graph angular coefficient changed when CA reached 100 μM. It is worth noticing that higher CA concentrations had no further effect on its antioxidant activity. This may suggest that CA is chelating Fe(II) in a 1:2 Fe(II):CA ratio, since higher CA concentrations had little influence on the DMPO-OH signal and on the 2-DR degradation. Noteworthy, since there is an excess of CA in solution regarding iron irons, the occurrence of more saturated complex species is also possible. For instance, CA could also form complexes with stoichiometry 1:3 Fe:CA, a more likely situation, since iron(II) ions prefer octahedral geometrical environments. Moreover, the behavior observed in Fig 3A could also be due to a saturation effect of the signal when the metal-ligand species are formed. Apparently, an increase in the iron concentration could change the pattern of the complexation, probably by the iron coordination on the other hydroxyl groups (see reference [25] for more details).

Observations from Ginani (2005) regarding the polyphenol ellagic acid (a molecule bearing two cathecol groups), showed that its antioxidant activity is pH-dependent against Fenton-induced oxidative damage [28]. Increasing the medium pH from 5.5 to 8.0 promoted a higher iron chelation and also an improvement in the antioxidant activity exhibited by the polyphenol. These observations taken as a whole indicate that in higher pHs the deprotonation of the cathecol groups is more elevated which, in turn, favors iron chelation, thus improving the capacity of CA to inhibit ROS formation. Altogether, these results strongly suggest that the mechanism for antioxidant action of CA is associated with the formation of the iron complex which, somehow, inhibits the reaction between hydroxyl radical and the target molecule (2-DR or DMPO).

The inverse relation between iron concentration and *in vitro* antioxidant action observed in Fig 3B was also reported for the case of TA, mangiferin and PIH [25,29,30]. These competition assays suggest that the inhibitory action on 2-DR degradation or DMPO hydroxylation was linked to the capacity of these molecules (including CA) to chelate iron ions. Thus, the higher the metal concentration in the media the lower is the ability of CA–and other iron chelating antioxidants–to form an iron-complex and prevent hydroxyl radical-mediated target oxidation. This indicates that the antioxidant capacity of CA is due to the formation of complexes with metal ions, capable of preventing in vitro oxidative damage.

In order to further characterize the antioxidant mechanism of CA an experiment varying the 2-DR concentration was performed (Table 1). This experiment allows the differentiation between the radical scavenger and the metal chelating mechanisms [31]. The scavenger mechanism is based on the reaction of the antioxidant directly with the hydroxyl radical, characterizing a competition system. Hence, an augment in the 2-DR concentration would cause a decrease in the percent of inhibition (of 2-DR degradation), since more hydroxyl radical would react more with the target molecule. However, if the antioxidant acts as chelator, complexing metal ions and preventing “free hydroxyl radical” formation, the percent of inhibition would not be altered when the target molecule concentration is increased. As shown in Table 1 the percent of inhibition remained unchanged in all 2-DR concentrations, a behavior strongly consistent with the metal chelator mechanism of antioxidant action. Similar conclusions were made by Hermes-Lima et al. (2000) [31], Andrade Jr et al. (2006) [23] and Pardo-Andreu et al. (2006) [25] when studying the antioxidant mechanisms of PIH, TA and mangiferin, respectively. On the other hand, incubations of Fe(II) plus H_2_O_2_ (6 μM and 100 μM, respectively) with “classical” scavenger molecules, such as mannitol and thiourea (both in milimolar concentrations), show that the percent of inhibition decreases with the augment in the 2-DR concentration (from 3 to 70 mM) [29]. This decrease in the percent of inhibition in the oxidation of a target molecule is a characteristic behavior of molecules bearing a scavenger antioxidant mechanism. It is worth mentioning that, despite several studies show that polyphenols, including CA, have antioxidant properties, this is the first study that clearly indicates that the antioxidant activity of this phenolic compound is influenced by the medium pH and its mechanism is related to its ability to form complexes with iron that prevent target oxidation by hydroxyl radicals. Despite many studies–including ours–showing that polyphenols act as antioxidant against metal-mediated free radical generation, some studies show the reverse effect [32]. For instance, Mauryan and Devasagayam (2010) reported that CA could act as a pro-oxidant species in systems containing Fe(III) ions, H_2_O_2_ and ascorbic acid. The authors attribute this capacity to a reducing ability of CA. In our conditions, however, we did not notice such behavior, which could be related to differences in the experimental conditions, including: ***i)*** we used Fe(II) instead of Fe(III); ***ii)*** there was no ascorbic acid in our incubations.

The antioxidant effect of CA against membrane lipid peroxidation was more pronounced than on the other systems, in which it prevented the peroxidation in lower concentrations (Fig 5A) (the other systems are based on assays for hydroxyl radical formation). This higher antioxidant activity could be due to the hydrophobicity of CA, which would increase its antioxidant activity in a lipophilic environment (e.g., cell membrane). In this system, CA could intercalate between the phospholipids present in biological membranes, acting as a peroxyl radical scavenger. Moreover, CA could chelate iron ions in the membrane microenvironment, preventing both initiation and propagation of lipid peroxidation. In this sense, CA prolonged the lag phase of lipid peroxidation in a dose-dependent manner (Fig 5B), suggesting a peroxyl radical scavenger mechanism [33]. This mechanism is well known for several molecules, including BHT, that are usually capable of donating a hydrogen atom to peroxyl radicals (for more details, see reference [34]). Moreover, CA also decreased the rate of the propagation phase of lipid peroxidation (log phase) in a dose-dependent manner (Fig 5C). This indicates that the antioxidant mechanism also involves iron complexation by CA, which prevents their participation in the propagation cascade.

These results altogether point toward a chelating mechanism of CA, in which it binds to iron ions and prevent the oxidative consequences of the Fenton reaction, including lipid peroxidation, DMPO hydroxylation and 2-DR damage. Since iron ions are mostly not “free” inside the cell (it is bound to proteins and low molecular weight ligands, such as citrate [35]), the antioxidant properties of CA reported herein might occur in the gastro-intestinal tract, a place where iron ions are highly absorbed. In this scenario, CA could act preventing oxidative damage promoted by iron and other transition metals. Moreover, studies analyzing the polyphenol concentration, including CA, in the blood of coffee drinking individuals indicated its presence in the low micromolar range [4,8,9], which gives it the capability to act in the cell membrane microenvironment of different tissues preventing lipid peroxidation and other oxidative processes mediated by lipid peroxidation products.

In addition to the antioxidant effect *per se*, evidences have been published indicating the effects of CA and its derivatives (for example, 3-(3,4-dihydroxy-phenyl)-acrylic acid 2-(3,4-dihydroxy-phenyl)-ethyl ester—CADPE) on gene expression [36]. Several studies reported the tumor suppression and potential therapeutic effects of CA *in vivo* [37]. For instance, Chung et al (2004) demonstrated that CA suppressed the tumor growth and liver metastasis in mice by down-regulating NF-kB and MMP-9 genes [36]. Indeed, such molecules were reported to down-regulate NF-kB in many cell-culturing experiments. Furthermore, it has been shown that CA and its derivatives are also able to enhance Nrf2 production, which would end up improving cellular antioxidant response [38–42]. Even though gene expression was out of the scope of our study, it is important to mention that both mechanisms—modulation of gene expression and antioxidant activity—may explain the beneficial health effects of CA.

In conclusion, our results strongly suggest that CA works *in vitro* by efficiently chelating iron ions and, thus, minimizing the effects of Fenton-generated hydroxyl radicals, including lipid peroxidation. Overall, we believe that the detailed chemical study presented here will expand the knowledge about the chemistry of caffeic acid, which might shed light on the biological functions of this molecule (as well as other phenolic compounds).
