# Correction: Antioxidant Activity of Caffeic Acid against Iron-Induced Free Radical Generation—A Chemical Approach

**DOI:** 10.1371/journal.pone.0142402

**Published:** 2015-11-03

**Authors:** Thiago C. Genaro-Mattos, Ângelo Q. Maurício, Daniel Rettori, Antonio Alonso, Marcelo Hermes-Lima

The Funding section is incorrect. The correct Funding information is as follows: This research was funded by grants from CNPq, Fundação de Amparo à Pesquisa do Distrito Federal (FAPDF—Grant193.000.040/2012), Universidade de Brasília and INCT de Processos Redox em Biomedicina.
